# Trace Mineral Supplementation for the Intestinal Health of Young Monogastric Animals

**DOI:** 10.3389/fvets.2019.00073

**Published:** 2019-03-13

**Authors:** Marcia Carlson Shannon, Gretchen Myers Hill

**Affiliations:** ^1^Division of Animal Science, University of Missouri, Columbia, MO, United States; ^2^Department of Animal Science, Michigan State University, East Lansing, MI, United States

**Keywords:** monogastric animal, intestinal health, nutrition, zinc, microorganisms

## Abstract

Growth performance and feed efficiency are essential parameters when evaluating profitability of livestock. However, animal performance does not always reflect optimal gut health. Decades of research have supported the theory that improved animal performance such as average daily gain and feed efficiency can be impacted by intestinal health or the ability of the intestinal mucosa to absorb nutrients, but dysfunction may be found when the animal is stressed. Most of the early research focused on enteric infections causing diarrhea and nutritional alternatives to antibiotics which has led to findings related to pharmacological supplementation of trace minerals above the nutrient requirements for non-ruminants. While pharmacological concentrations of copper (Cu) have been shown to enhance growth, the mechanism in the gut is elusive. High concentrations of zinc (Zn) fed to newly weaned nursery pigs reduced the incidence of diarrhea from the proliferation of enterotoxigenic *Escherichia coli* (*E. coli*) and *Clostridium* and improve gut morphology. There are numerous publications where pharmacological supplementation of Zn as zinc oxide (ZnO) were fed to newly weaned pigs. Pharmacological Zn has been reported to shape the intestinal microflora as well as the diversity of the microflora during the first 2 weeks post-weaning. Both Fe deficiency and fortification impact bacterial growth in the intestine. Therefore, this paper will focus on the role of trace minerals that potentially impact optimal gut health of young monogastric animals.

## Introduction

Monogastric animal production and profitability have relied on genetic improvements, meeting nutritional requirements, and animal health in order to maximize growth performance and ensure improved efficiency. For both pig and broiler production, producers have been working for decades to increase efficiency and reduce the cost of production. Siegel (1) reported that broiler feed conversion ratio from 1985 to 2010 has improved by almost 35% from 2.3 to 1.5, respectively. Similarly, data (2) from the swine industry support an improvement in feed conversion from 3.6 to 2.7 between 1986 and 2016. However, it is believed that there are still improvements to be made based on genetic potential and nutritional interventions associated with intestinal microflora of monogastric animals. The diversity of microbiome is important in digestion of animal feeds in the gastrointestinal tract. This was demonstrated by Frese et al. (3) and Bian et al. (4), who characterized the fecal microbiome of nursing or weanling pigs and determined that gut microbial populations are clearly established based on dietary content consumed. De Rodas et al. (5) recently reported the bacterial microbiome throughout the gastrointestinal tract from birth to market weight to have increased Clostridia and a decreased Gammas proteobacteria with age. Hence, reminding us that the microbiome is not just influenced by feedstuffs but age.

## Industry Management Issues

The swine industry is continuing to evaluate herd efficiency and health as related to standard operating procedures. The weaning period on a swine farm is probably the most stressful phase for the young pig which often results in disruptive intestinal barrier integrity due to enterotoxigenic infectious such as *Escherichia coli*, and ultimately a reduction in growth performance for the first few days post-weaning often referred to a post-weaning lag. Moeser et al. (6) reported that weaned pigs at an older age (22–24 d of age) were less likely to have an exacerbated immune response from an *E. coli* challenge when compared to pigs weaned at a younger age (16–18 d of age). Therefore, early-weaned pigs that are stressed during the weaning process may have an altered intestinal microflora and permeability. Additionally, Moeser (7) has reported that the sex of the animal, as well as, weaning age affects gut health; therefore, possibly influencing the permeability of the intestinal lining. He notes that it will “limit or tightly regulate the exposure of environmental antigens (e.g., feed antigens), toxins, and microorganisms to the gut mucosal immune system” (7). At 24 h post-weaning, De Rodas et al. (5) found that relative abundance of Lactobacillaceae was reduced. As important, the introduction of feed from 21 to 33 days of age had more effect on the microbiome than age, changes in type of feed or change in location for the entire study.

Therefore, in order for the swine industry to overcome the weaning stress issues, much research has evaluated the impact of weaning age and weaning weights on growth performance of nursery pigs. Feed additive antibiotics have historically been used to control pathogenic infections in swine production. However, culturally, antibiotic usage for general control of pathogens have been more closely regulated in order to curtail the antibiotic resistance in the human populations (8). Therefore, finding nutraceutical feedstuffs that can alter gut health and also improve animal performance is essential. Since the challenge of today's modern hog operations who maintain an antibiotic free system is the prevention of gastrointestinal disease especially when the production is located in high swine concentration environment.

The intestinal microbiota of young piglets can adapt quite quickly to dietary changes (liquid diet to a solid diet) or other environmental stresses. During this critical time, intestinal pathogens often thrive resulting in observations of diarrhea in newly weaned pigs. So, any nutritional interventions through trace mineral supplementation to alleviate post-weaning diarrhea of newly weaned pigs will ultimately improve gut health and overall animal well-being.

Unlike pig production, broiler production does not have a post-weaning lag period associated with weaning the piglet from the sow, but post-hatch chicks often experience a delay in access to feed and water due to the time between hatching and delivery to the farm. The yolk sac functions as an internal nutritional reserve, but often newly hatched chicks will have decreased growth performance, gastrointestinal development, and immunosuppression due to water and feed deprivation (9–11). Zinc is an essential trace mineral for poultry and required for normal growth, reproduction and development of the enzymatic systems (12). The supplementation of dietary Zn has subsequently been introduced into broiler chickens and has been shown to alleviate the loss of intestinal mucosal barrier function (13). Hu et al. (14) concluded that Zn supplemented at 60 ppm improved growth, intestinal micro-flora, gut morphology, and barrier function in broilers.

## Copper and Zinc

The biological necessity of trace minerals for growing animals was determined many years ago (15). However, researchers continue to determine the mechanism and impact of their interactions on performance as well as intestinal microbial population. Emphasis in this area of research has increased in recent years due to the increased concern of antibiotic use in livestock production and the need to find alternatives to antibiotics.

The Cu requirement for young nursery pigs weighing 5–20 kg has been established as 5 to 6 mg/kg/d (16). Copper has been reported to provide antimicrobial effects when fed above the requirement in pharmacological concentrations (100–250 ppm). Currently, there is no agreement relative to the mechanism involved, but Shurson et al. (17) reported an acceleration in intestinal cell turnover while Zhao et al. (18) found reduced villus height. Interestingly early work showed that copper sulfate stimulates average daily gain in nursery pigs (19–21) and today many forms of Cu are effective in stimulating gain Spears laboratory (22) reported that the villus height was greater in the duodenum and reduced in jejunum in pigs fed 225 mg Cu as sulfate compared to control pigs (6.7 ppm Cu). Additionally, duodenal malondialdehyde concentrations followed this same pattern. However, hepatic cytochrome c oxidase assembly protein 17 mRNA was less and expression of antioxidant l mRNA greater in the sulfate supplemented pigs compared to the controls. Pigs fed 225 Cu as sulfate or tribasic Cu chloride (TBCC) had greater Cu concentrations in the mucosa of the small intestine than pigs fed 5 ppm Cu., and sulfate resulted in higher concentrations in the mucosal duodenum than pigs fed TBCC. The mRNA of duodenal antioxidant 1 was downregulated in sulfate pigs compared to the control pigs while hepatic Cu transporting β-polypeptide ATPase was upregulated in pigs fed 225 ppm Cu vs. controls (23).

These new laboratory techniques may yield information relative to the mechanism(s) involved in performance enhancement via trace mineral supplementation. However, there is not a consistent response to pharmacological Cu suggesting that various mechanisms and interactions on gut health may be involved.

Pharmacological Cu is frequently fed during the early part of the grow/finish phase at 150–200 ppm. It is interesting to note that in the first dietary phase of grow/finish (44 days post weaning; 63 days of age) the dominant operational taxonomic unit classified as Campylobacter were negatively correlated with body weight in the De Rodas et al. (5) study while being fed 150 ppm Cu that followed 200 ppm Cu in all nursery diets. Campylobacter is a commensal bacterium that is present in pigs at most ages.

Zinc is an essential nutrient for normal development, growth, DNA synthesis and many cellular functions, and the zinc requirement for young nursery pigs weighing 5–20 kg has been established as 80–100 mg/kg/d (16). However, many researchers further evaluating the 3,000 ppm Zn supplementation work by Poulsen (24) observed that higher concentrations of Zn as ZnO may improve young pig growth performance (25) as well as reduce scouring (26, 27). Carlson et al. (28) and Case and Carlson (29) determined that the feeding of pharmacological concentrations of Zn in the form of ZnO at a rate of 3,000 mg/kg/d improves growth performance of newly weaned nursery pigs. The observed improvement in growth performance has mainly been attributed to the decrease in the presence of *Escherichia coli* bacteria count (30). Mechanistically, Carlson et al. (28) observed alternations in the duodenum such as deeper crypts and greater total thickness, as well as increased intestinal metallothionein (MT) concentrations in nursery pigs fed diets supplemented with 3,000 ppm Zn as ZnO. Thus, her work indicates that high concentrations of Zn have an impact on intestinal health. Based on knowledge from human medical research, animal research looked closer as the intestinal pre-infection microorganisms and a decrease in diversity of the gut microbiome after weaning (31). High concentrations of dietary ZnO have been shown to be beneficial for maintaining the stability of the intestinal microflora, to support a large diversity of coliforms in weaned pigs (32), and to reduce the susceptibility of the pigs to *Escherichia coli* infections (33). Li et al. (34) confirmed these findings when feeding 3,000 ppm Zn as ZnO to 21 d-old weaned pigs resulting in increased mucosal thickness and villous width of the small intestine.

Vahjen et al. (35) determined that 40–42 d old pigs showed no changes in the order level of ileal microbiome when fed 3,000 ppm Zn as ZnO, but genus level changes were observed. For example, *Streptococcus* increased while *Sarcina* decreased. In addition, Li et al. (34) reported no effect of pharmacological concentrations of Zn as ZnO supplementation for nursery pigs on the number of *Enterobacteriaceae, Clostridia*, and *Lactobacilli* in ileal digesta and feces. In contrast, Broom et al. (36) and Jensen-Waern et al. (37) found that pharmacological concentrations of Zn as ZnO reduce fecal counts of *Lactobacilli* and *enterococci* during the post-weaning period of pigs, but only temporarily. In agreement, Hojberg et al. (38) reported that feeding weaned piglets 2,500 ppm of Zn as ZnO reduced the MRS counts (lactic acid bacteria) and Rogosa counts (*lactobacilli*) for all segments of the gastrointestinal tract. Impact of ZnO fed at pharmacological concentrations on the microbiome cannot be determined in the De Rodas et al. (5) research since 3,500 was fed the first 8 days after weaning and 2,000 was fed from days 8 to 22 post weaning to all pigs.

Studies have shown that *Lactobacilli* are considered to have beneficial effects on human and animal health (39, 40) due to its antimicrobial activity against microbial pathogens (41). *Lactobacilli* are among the earliest bacteria to colonize the gut (41). The populations of *Lactobacillus* are thought to be found in high populations in weaned pigs. However, De Rodas et al. (5) reported that *Lactobacillaceae* were reduced 24 h after weaning. Studies *in vitro* and in animals have shown that *lactobacilli* may prevent *Escherichia coli* from colonizing in the jejunum and produce substances directed against the enterotoxins resulting in an inhibition of *Escherichia coli*-induced enterotoxin reactions (42–44). In agreement, Conway (45) and Chan et al. (46) studying the concept of competitive exclusion of pathogenic *Escherichia coli* by lactobacilli in the intestine and urinary tract *in vitro*, respectively, found that the colonization of the *lactobacilli* sterically hindered the adhesion of *Escherichia coli* to the surface. Importantly, Sawai (47) reported ZnO inhibits *Staphylococcus aureus* and *Escherichia coli* growth in the intestine. Thus, providing another mode of action of ZnO fed in pharmacological concentrations.

Roselli et al. (48) using cell culture techniques reported that ZnO may protect intestinal cell from *Escherichia coli* infections by inhibiting the adhesion and internalization of bacteria, preventing the disruption of barrier integrity, and modulating cytokine gene expression, but not by a direct antibacterial effect. Feed grade sources of ZnO vary in color, texture, content, and processing method. Also, ZnO sources tested by chick assays ranged in bioavailability from 37 to 93% based on weight gain and tibia Zn (49). Mavromichalis et al. (50) observed that nursery pigs fed ZnO sources with either high (95%) or low (35%) bioavailability did not affect the growth performance and gut morphology during the entire 21-d assay.

Olukosi et al. (51) reported in broilers that form and amount of Cu and Zn affected performance, percent of breast meat, and concentration of hepatic Cu. As seen by Carlson et al. (28) in pigs, villus height and the villus height to crypt depth ratio were higher in the duodenum when broilers were fed a ZnO source. This data suggests less intestinal permeability when utilizing *ex-vivo* Using chambers; Hu et al. (14) observed reduced colonic permeability to mannitol and inulin.

In the pre-ruminant calf, Jenkins and Hidiroglou (52) showed that 700 and 1,000 ppm Zn reduced weight gain, feed intake and efficiency compared to intakes from 40 (NRC recommendations) to 500 ppm. Dosing calves with 40 g Zn/d resulted in neonatal calves recovering from diarrhea 1 day earlier than controls (53). Perhaps these findings are a result of the observations of Rodriguez et al. (54) in guinea pigs and shigellosis infected children (55) that the increased intestinal paracellular permeability observed during fasting and malnutrition is prevented by pharmacological Zn. Additionally, Zn has been reported to promote epithelialization and anti-infective in wound healing (56).

The phytate component in feed ingredients is reported to affect Zn solubility and gut pH (57). Additionally, when pharmacological Zn and Cu are fed, the Zn: phytate and Cu: phytate ratios are altered. Soluble complexes are present at intestinal pH values when the ratio exceeds 10:1, but insoluble complexes are formed when more than one divalent cation per phytate is formed. Hence, the observed effect of pharmacological Zn and or Cu on phytase activity (58) may explain the observed variation in performance outcomes when pharmacological Zn or Cu is fed to pigs. In broilers, Morgan et al. (59) reported that phytase activity impacted Zn concentration in the gizzard and ileum but not the duodenum indicating the importance of not making assumptions on Zn metabolism between species.

Hill's laboratory (60–62) has explored many of the Cu and Zn enzyme's activity and associated gene expression, but few swine and poultry researchers have determined Zn and Cu biological signals (63) such as transporters even though it has been shown in humans that dietary Zn intake influences Zn transporters in the plasma membrane of the intestine (64).

## Other Impacts on Intestinal Health

The physical process of weaning pigs from the sow regardless of piglet age has been characterized as the decrease in intestinal barrier function of the gut in the newly weaned pig resulting in decrease pig performance caused by increased intestinal permeability creating alternations in intestinal microbial populations as well as inflammation (65). There are several layers of protection in the intestinal lining against pathogens and toxins. The protection is very important during weaning in a baby pig's life due to the physiological stress of separation from the sow and converting to a dry grain based diet. During weaning, villus height decreases and the intestinal lining becomes more susceptible to pathogens causing reduction in growth performance through lower nutrient absorption. A practical solution for the swine industry for years has been using feed-grade antibiotics; however, more recently other dietary nutritional interventions have been researched such as feeding higher concentrations of trace minerals zinc and/or copper.

As noted earlier, Zn has been known to be essential in many biological functions of mammals, such as anti-inflammation, anti-diarrhea, and maintaining epithelial barrier integrity (48, 66). Feeding pigs' dietary concentrations of Zn as ZnO greater than the requirement has shown to impact intestinal morphology of growing pigs (28). Subsequent research supports similar findings with feeding Zn to growing pigs improves intestinal microflora and barrier function (67). Intestinal counts of *Clostridium* and *Escherichia coli* in the intestinal segment of the jejunum decreased linearly when nursery pigs were fed dietary concentrations of zinc (67).

Heat, crowding (68), and weaning are stressors (69) known to negatively impact intestinal health, feed consumption and weight gain. Sanz Fernandez et al. (70) reported that pigs exposed to 36°C with ~50% humidity demonstrated increased ileal and colonic permeability that was decreased by feeding a diet containing 220 ppm Zn. It is not clear if the effect on the gut health is totally due to reduced feed consumption, change in diet form, or decreased energy because husbandry practices often confound the research findings (71, 72).

## Iron and Manganese

The Fe requirement for young nursery pigs weighing 5–20 kg has been established as 100 mg/kg/d (16). Iron is an essential nutrient needed for hemoglobin in red blood cells where most of the body's Fe is found. However, newborn pigs are unique because of their rapid growth and low concentration of Fe in milk and hence need supplementation of 100–200 mg of injectable Fe in the first 3 days of life (19). Before gut closure, these newborn pigs can very efficiently absorb Fe from the intestinal mucosa (73). Oral Fe within the first few hours of life can be absorbed, but must be administered before gut closure to large molecules. Interesting, research with humans suggests that there is a lack of Fe homeostasis in young infants (74). In rats before weaning, pups cannot regulate Fe homeostasis regardless of Fe status, but 10 days later Fe transporters (DMT1 and ferroportin) in small intestine were affected by Fe status. There is no data to suggest if this occurs in pigs and poultry.

Lactoferrin is absorbed across the intestinal cell via the lactoferrin receptor and is found in colostrum, milk, saliva, tears, and nasal secretion as part of the immune system. Its roles include antimicrobial, immunomodulatory, and Fe binding. The number of eosinophils is higher in the intestine of healthy pigs. The migration of eosinophils, which increase during inflammation, is inhibited by lactoferrin. The receptors of lactoferrin in the pig are found on the duodenal brush border of villi, crypt, and within the lamina propria (75) Hence, this important Fe binding protein not only is important in Fe absorption in the duodenum but in controlling bacteria in the gut. The human small intestinal lactoferrin receptor has been cloned in the pig (76).

Iron toxicity has been shown to occur at 600 mg/kg of Fe in 3–10 d old pigs. In addition, growing-finishing pigs only require 40–50 mg/kg of Fe, but typically growing pig diets will contain four to five times more Fe than required. The excessive concentrations of Fe in commercial swine diets, that may be unavailable to the animal, is due to differing Fe bioavailability of Fe in dietary ingredients such as blood meal, dicalcium phosphate, and limestone (16, 77).

There is an interdependency of the transport mechanisms and regulation of Mn and Fe, two transition elements. Both utilize transporters especially divalent metal transporter-1 (DMT-1). Mammals will have abnormal accumulation of Mn when Fe is low in the diet/body and if Mn is excessive or low, Fe homeostasis is altered. For example, Hansen et al. (78) reported that pigs fed high Fe diets had lower gene expression of Fe encoded proteins in the liver (Hepcidin) and duodenum (DMT1) as well as Mn was lower in liver and greater in duodenum. These results indicate that dietary Fe supplementation may impair absorption of Mn, but not Cu and Zn.

The acidic environment of the stomach and effect of diet usually result in the Fe that reaches the stomach to be in the ferrous form, but as the pH increases and ferric Fe solubility decreases. Most Fe is absorbed in the duodenum, but the remainder goes to the colon where it is utilized by bacteria. There is the potential for limited Fe absorption from the colon. Rats treated with antibiotics had decreased absorption of Fe (79).

Iron is required for most bacteria to flourish since it serves as a co-factor in re-dox reactions, metabolic pathways and of course the electron transport chain reactions. A few bacteria such as *Borrelia burgdorferi* that causes Lyme disease use Mn instead in proteins requiring Fe. Most bacteria have increased viability in the presence of Fe, and an excess is believed to exacerbate most gut infections.

The research data associated with iron supplementation and the impact on gut health is inconclusive at best. Iron is in abundance and often not considered deficient or limiting in today's animal production. There is research that supports Fe supplementation and increases the presence of beneficial microbiota that may improve the overall gut health of the animal (80, 81). However, in a review, Lönnderdal (82) reminds us that excessive Fe has a negative effect on growth and microbial health of the gut if animals or humans had adequate Fe before supplementation. There was an increase in the proportion of anaerobes except when Fe supplementation was high. It is thought to occur because high Fe results in an increase in free reactive Fe that induces free radical damage in the gastrointestinal track by release of oxygen by Haber-Weiss reaction. This increase in oxidative stress will decrease the strict anaerobes.

The interaction of Fe and Mn may be the most important information that is known about Mn on gut health. In humans and experimental animals, it has been shown that Mn is not well-absorbed, and it appears from experimental animal studies that fiber, phytic acid, oxalic acid, Ca and P reduce its availability for absorption (83). Perhaps more importantly, Mn and Fe compete for binding sites that effects absorption and ultimately body stores (84).

It is believed that Mn absorption in poultry is less than pigs, but there is no definitive research with today's genetics in either species. Using polarographic analysis, solubility in buffers, and deionized water, Li et al. (85) reported that bioavailability of five different organic Mn sources was closely related to chelation strength. While organic Mn has been promoted to be of value in preventing leg abnormalities, this incidence of swelling of the tibia-tarsal joint abnormality is not prevented by Mn dietary supplementation. This broiler problem is more severe if poly-unsaturated fatty acids are added to the diet for growth promotion.

When inorganic and chelated minerals (Cu, Zn, Fe, Mn) fed at reduced dietary concentrations were compared to control fed pigs from weaning to finishing, reduced mineral concentrations regardless of source did not affect performance but resulted in reduced fecal excretion (86). Besides the role of Mn in metabolic functions of enzymes, supplementation of Mn has been reported to result in greater lean color scores and more vivid red color in pork chops when provided as a sulfate but not an organic form (87). This might indicate that absorption differed perhaps due to valence changes.

## Conclusion

Health of the gut is reflected in the performance of the animal from hatch/birth to death. Additionally, changes in the gut's morphology may not provide a means of evaluation of gut health. However, it is currently being used with the *ex vivo* studies with Using Chambers that determine the capacity of the intestine to transport nutrients. Hence, the techniques and technologies currently being used in research today will give additional information lacking in today's literature. Clearly, the published National Research Council's nutrient requirements for specific species should only be used a guidelines. However, researchers have determined that any disruption of the gastrointestinal tract will impact animal performance, and supporting the antioxidant system by the usage of trace mineral supplementation will ensure intestinal microbiota health and repair. In conclusion, much of the results of positive and negative attributes of trace mineral supplementation on gut health are influenced by genetics, production goals, and the environment.

## Author Contributions

All authors listed have made a substantial, direct and intellectual contribution to the work, and approved it for publication.

### Conflict of Interest Statement

The authors declare that the research was conducted in the absence of any commercial or financial relationships that could be construed as a potential conflict of interest.

## References

[1] Siegel PB. Evolution of the modern broiler and feed efficiency. Ann Rev Anim Biosci. (2014) 2:375–85. 10.1146/annurev-animal-022513-114132 25384148

[2] Stalder KJ Pork Industry Productivity Analysis. National Pork Board Research Grant Report. (2016). p. 1–16. Available online at: https://www.pork.org/wp-content/uploads/2017/11/2017-pork-industry-productivity-analysis.pdf

[3] Frese SA Parker K Calvert CC Mills DA. Diet shapes the gut microbiome of pigs during nursing and weaning. Microbiome. (2015) 3:28–38. 10.1186/s40168-015-0091-8 26167280 PMC4499176

[4] Bian G Ma S Zhu Z Su Y Zoetendal EG Mackie R. Age, introduction of solid feed and waning are more important determinants of gut bacterial succession in piglets than breed and nursing mother as revealed by a reciprocal cross-fostering model. Environ Microbiol. (2016) 18:1566–77. 10.1111/1462-2920.13272 26940746

[5] De Rodas B Youmans BP Danzeisen JL Tran H Johnson TJ. Microbiome profiling of commercial pigs from farrow to finish. J Anim Sci. (2018) 96:1778–94. 10.1093/jas/sky109 29635455 PMC6140882

[6] Moeser AJ Pohl CS Rajput M. Weaning stress and gastrointestinal barrier development: implications for lifelong gut health in pigs. Anim Nutr. (2017) 3:313–21. 10.1016/j.aninu.2017.06.003 29767141 PMC5941262

[7] Moeser AJ Gender and stress matter in pig gut health. National Hog Farmer Daily (2018, April 17).

[8] Teillant A Brower CH Laxminarayan R Economics of antibiotic growth promoters in livestock. Ann Rev Resour Econ. (2015) 7:349–74. 10.1146/annurev-resource-100814-125015

[9] Ao Z Kocher A Choct M. Effects of dietary additives and early feeding on performance, gut development and immune status of broiler chickens challenged with clostridium perfringens. Asian Aust J Anim Sci. (2012) 25:541–51. 10.5713/ajas.2011.11378 25049595 PMC4092898

[10] Decuypere E Tona K Bruggeman V Bamelis E The day-old chick: a crucial hinge between breeders and broilers. World's Poult Sci J. (2001) 57:127–38. 10.1079/WPS20010010

[11] Dibner JJ Knight CD Kitchell ML Atwell CA Downs AC Ivey FJ Early feeding and development of the immune system in neonatal poultry. J Appl Poult Res. (1998) 7:425–36. 10.1093/japr/7.4.425

[12] NRC Nutrient Requirements of Poultry, 9th ed Washington, DC: National Academy of Sciences.

[13] Zhang BY Shao Y Liu D Yin P Guo Y Yuan J. Zinc prevents *Salmonella enterica* serovar Typhimurium-induced loss of intestinal mucosal barrier function in broiler chickens. Avian Pathol. (2012) 41:361–7. 10.1080/03079457.2012.692155 22834550

[14] Hu CH Qian ZC Song J Luan ZS Zuo AY. Effects of zinc oxide-montmorillonite hybrid on growth performance, intestinal structure, and function of broiler chicken. Poult Sci. (2013) 92:143–50. 10.3382/ps.2012-02250 23243241

[15] Cousins RJ Clinical, Biochemical, and Nutritional Aspects of Trace Elements. New York, NY: Alan R. Liss Inc. (1982). p. 117.

[16] NRC (2012). Nutrient Requirements of Swine, 11th ed Washington, DC: National Academy of Sciences.

[17] Shurson GC Ku PK Waxler GL Yokoyama MT Miller ER. Physiological relationships between microbiological status and dietary copper levels in the pig. J Anim Sci. (1990) 68:1061–71. 10.2527/1990.6841061x 2332383

[18] Zhao J Harper AF Estienne MJ Webb KE Jr McElroy AP Denbow DM. Growth performance and intestinal morphology responses in early weaned pigs to supplementation of antibiotic-free diets with an organic copper complex and spray-dried plasma protein in sanitary and nonsanitary environments. J Anim Sci. (2007) 85:1302–10. 10.2527/jas.2006-434 17264238

[19] Barber RS Braude R Mitchell KG Cassidy J High copper mineral mixtures for fattening pigs. Chem Ind. (1955) 21:601–3.

[20] Bunch RJ McCall JT Speer VC Hays VW Copper supplementation for weanling pigs. J Anim Sci. (1965) 24:995–9. 10.2527/jas1965.244995x

[21] Braude R Some observations on the need for copper in the diet of fattening pigs. J Agr Sci. (1945) 35:163–7. 10.1017/S0021859600049170

[22] Fry RS Ashwell MS Lloyd KE O'Nan AT Flowers WL Stewart KR. Amount and source of dietary copper affects small intestine morphology, duodenal lipid peroxidation, hepatic oxidative stress, and mRNA expression of hepatic copper regulatory proteins in weanling pigs. J Anim Sci. (2012) 90:3112–9. 10.2527/jas.2011-4403 22585802

[23] Huang YL Ashwell MS Fry RS Lloyd KE Flowers WL Spears JW. Effect of dietary copper on stress of weanling pigs in short-term feeding. J Anim Sci. (2015) 93:2948–55. 10.2527/jas.2014-8082 26115281

[24] Poulsen HD Zinc Oxide for Pigs During Weaning. Dublin: EAAP - European Association of Animal Production (1989).

[25] Hahn JD Baker DH Growth and plasma zinc responses of young pigs fed pharmacological levels of zinc. J Anim Sci. (1993) 71:3020–4. 10.2527/1993.71113020x 8270523

[26] Rutkowska-Pejsak B Mokrzycka A Szkoda J Influence of zinc oxide in feed on health status of weaned pigs. Medycyna Weterynaryjna. (1998) 54:194–200.

[27] Heo JM Kim JC Hansen CF Mullan BP David BH Maribo H Effects of dietary protein level and zinc oxide supplementation on the incidence of post-weaning diarrhea in weaner pigs challenged with an enterotoxigenic strain of *Escherichia coli*. Livestock Sci. (2010) 133:210–3. 10.1016/j.livsci.2010.06.066

[28] Carlson MS Hill GM Link JE. Early and traditionally weaned nursery pigs benefit from phase-feeding pharmacological concentrations of zinc oxide: effect on metallothionein and mineral concentrations. J Anim Sci. (1999) 77:1199–207. 10.2527/1999.7751199x 10340587

[29] Case CL Carlson MS. Effect of feeding organic and inorganic sources of additional zinc on growth performance and zinc balance in nursery pigs. J Anim Sci. (2002) 80:1917–24. 10.2527/2002.8071917x 12162660

[30] Holm A Poulsen HD Zinc oxide in treating *E*. coli diarrhea in pigs after weaning. Comp Cont Ed Pract Vet. (1996) 18:S26–9.

[31] Guevarra RB Hong SH Cho JH Kim B Shin J Lee JH. They dynamics of the piglet gut microbiome during the weaning transition in association with health and nutrition. J Anim Sci Biotech. (2018) 9:54–63. 10.1186/s40104-018-0269-6 30069307 PMC6065057

[32] Katouli M Melin L Jensen-Waern M Wallgren P Mollby R. The effect of zinc oxide supplementation on the stability of the intestinal flora with special reference to composition of coliforms in weaned pigs. J Appl Microbiol. (1999) 87:564–73. 10.1046/j.1365-2672.1999.00853.x 10583685

[33] Mores N Christani J Piffer IA Barioni W Lima GMM Effects of zinc oxide on postweaning diarrhea control in pigs experimentally infected with *E*. coli. Arg Brasil Med Vet Zootec. (1998) 50:513–23.

[34] Li BT Van Kessel AG Caine WR Huang SX Kirkwood RN Small intestinal morphology and bacterial populations in ideal digesta and feces of newly weaned pigs receiving a high dietary level of zinc oxide. Can J Anim Sci. (2001) 81:511–6. 10.4141/A01-043

[35] Vahjen W Pieper R Zentek J. Bar-coded pyrosequencing of 16s rRNA gene amplicons reveals changes in illeal porcine bacterial communities due to high dietary zinc intake. Appl Environ Microbiol. (2010) 76:6689–91. 10.1128/AEM.03075-09 20709843 PMC2950476

[36] Broom LJ Miller HM Kerr KG Toplis P Removal of both zinc oxide and avilamycin from the post-weaning piglet diet: consequences for performance through to slaughter. Anim Sci. (2003) 77:79–84. 10.1017/S1357729800053674

[37] Jensen-Waern M Melin L Lindberg R Johannisson A Petersson L Wallgren P. Dietary zinc oxide in weaned pigs-effects on performance, tissue concentrations, morphology, neutrophil functions and faecal microflora. Res Vet Sci. (1998) 64:225–31. 10.1016/S0034-5288(98)90130-8 9690608

[38] Hojberg O Canibe N Poulsen HD Hedemann MS Jensen BB. Influence of dietary zinc oxide and copper sulfate on the gastrointestinal ecosystem in newly weaned piglets. Appl Environ Microbiol. (2005) 71:2267–77. 10.1128/AEM.71.5.2267-2277.2005 15870311 PMC1087531

[39] Fuller R Probiotics. The Scientific Basis. London: Chapmann and Hall (1992). pp. 398.

[40] Sanders ME. Effect of consumption of lactic cultures on human health. Adv Food Nutr Res. (1993) 37:67–130. 10.1016/S1043-4526(08)60116-3 8398048

[41] Servin AL. Antagonistic activities of lactobacilli and bifidobacteria against microbial pathogens. FEMS Microbiol Rev. (2004) 28:405–40. 10.1016/j.femsre.2004.01.003 15374659

[42] Foster TL Winans L Jr Carski TR. Evaluation of lactobacillus preparation on eterotoxigenic *E*. coli-induced rabbit ileal loop reactions. Am J Gastroenterol. (1980) 73:238–43. 6996476

[43] Johnson DE Calia FM The effect of Lactinex on rabbit ileal loop reactions induced by enterotoxigenic Escherichia coli. Curr Microbiol. (1979) 2:207–10.

[44] De Mitchell IG Kenworthy R Investigations on a metabolite from *Lactobacillus bulgaricus* which neutralizes the effect of enterotoxin from *Escherichia coli* pathogens for pigs. J Appl Bacteriol. (1976) 41:163–8. 10.1111/j.1365-2672.1976.tb00615.x 783106

[45] Conway PL Lactobacilli: fact and fiction. In: Grubbe R Midtvedt T Norin E editors. The Regulatory and Protective Role of the Normal Microflora. New York, NY: MacMillan Press (1989). pp. 263–81.

[46] Chan RCY Reid G Irwin RT Bruce AW Costerton JW. Competitive exclusion of uropathogens from human uroepithelial cells by *Lactobacillus* whole cells and cell wall fragments. Infect Immun. (1985) 47:84–9. 3917428 10.1128/iai.47.1.84-89.1985 PMC261473

[47] Sawai J. Quantitative evaluation of antibacterial activities of metallic oxide powders (ZnO, MgO and CaO) by conductimetric assay. J Microbiol Methods. (2003) 54:177–82. 10.1016/S0167-7012(03)00037-X 12782373

[48] Roselli M Finamore A Garaguso I Britti MS Mangheri E. Zinc oxide protects cultured enterocytes from the damage induced by Escherichia coli. J Nutr. (2003) 133:4077–82. 10.1093/jn/133.12.4077 14652351

[49] Edwards HM III Boling SD Emmert JL Baker DH. Bioavailability of zinc in two zinc sulfate by-products of the galvanizing industry. Poultry Sci. (1998) 77:1546–9. 10.1093/ps/77.10.1546 9776064

[50] Mavromichalis I Peter CM Parr TM Ganessunker D Baker DH Growth-promoting efficacy in young pigs of two sources of zinc oxide having either a high or a low bioavailability of zinc. J Anim Sci. (2000) 78:2896–902. 10.2527/2000.78112896x 11063314

[51] Olukosi OA van Kuijk S Han Y. Copper and zinc sources and levels of zinc inclusion influence growth performance, tissue trace mineral content, and carcass yield of broiler chickens. Poultry Sci. (2018) 97:3891–8. 10.3382/ps/pey247 29982614 PMC6162356

[52] Jenkins KJ Hidiroglou M Tolerance of the preruminant calf for excess manganese or zinc in milk replacer. J Dairy Sci. (1991) 74:1047–53. 10.3168/jds.S0022-0302(91)78254-4 2071705

[53] Glover AD Puschner B Rossow HA Lehenbauer TW Champagne JD Blanchard PC. A double-blind block randomize clinical trial on the effect of zinc as a treatment for diarrhea in neonatal Holstein calves under natural challenge conditions. Prev Vet Med. (2013) 112:338–47. 10.1016/j.prevetmed.2013.09.001 24074841 PMC7114245

[54] Rodriguez P Darmon N Chappuis P Candalh C Blaton MA Bouchaud C Intestinal paracellular permeability during malnutrition in guinea pigs: effect of high dietary zinc. Gut. (1996) 9:416–22. 10.1136/gut.39.3.416 PMC1383349 8949647

[55] Alam AN Sasrker SA Wahed MA Khatun M Abdur Rahaman M. Enteic protein loss and intestinal permeability changes in children during acute shigellosis and after recovery: effect of zinc supplementation. Gut. (1994) 35:1707–11. 10.1136/gut.35.12.1707 7829006 PMC1375257

[56] Landsdown AB Mirastschijski U Stubbs N Scanlon E Ågren MS Zinc in wound healing: theoretical, experimental, and clinical aspects. Wound Repair Regen. (2007) 15:2–16. 10.1111/j.1524-475X.2006.00179.x 17244314

[57] Selle PH Ravindran V Phytate-degrading enzymes in pig nutrition. Livest Sci. (2008) 113:99–122. 10.1016/j.livsci.2007.05.014

[58] Martinez MM Link JE Hill GM. Dietary pharmacological or excess zinc and phytase effects on tissue mineral concentrations, metallothionein, and apparent mineral retention in the newly weaned pig. Biol Trace Elem Res. (2005) 105:97–116. 10.1385/BTER:105:1-3:097 16034157

[59] Morgan NK Scholey DV Burton EJ. Use of Zn concentration in the gastrointestinal tract as a measure of phytate susceptibility to the effect of phytase supplementation in broilers. Poult Sci. (2017) 96:1298–305. 10.3382/ps/pew394 27789748

[60] Rincker MJ Clarke SL Eisenstein RS Link JE Hill GM Effects of Fe supplementation on binding activity of iron regulatory proteins and the subsequent impact on growth performance and indices of hematological and mineral status of young pigs. J Anim Sci. (2005) 83:2137–45. 10.2527/2005.8392137x 16100069

[61] Martínez-Montemayor MM Hill GM Raney NE Rilington V Tempelman RJ Link JE. Gene expression profiling in hepatic tissue of newly weaned pigs fed pharmacological zinc and phytase supplemented diets. BMC Genomics. (2008) 9:421. 10.1186/1471-2164-9-421 18799003 PMC2566318

[62] Martin RE Mahan DC Hill GM Link JE Jolliff JS. Effect of dietary organic microminerals on starter pig performance, tissue mineral concentrations, and liver and plasma enzyme activities. J Anim Sci. (2011) 89:1042–55. 10.2527/jas.2009-2384 21415420

[63] Cousins RJ Liuzzi JP Lichten LA. Mammalian zinc transport, trafficking, and signals. J Biol Chem. (2006) 281:24085–9. 10.1074/jbc.R600011200 16793761

[64] Cragg RA Phillips SR Piper JM Varma JS Campbell FC Mathers JC. Homeostatic regulation of zinc transporters in the human intestine by dietary zinc supplementation. Gut. (2008) 54:469–78. 10.1136/gut.2004.041962 15753530 PMC1774437

[65] Blikslager AT Moeser AJ Gookin JL Jones SL Odle J. Restoration of barrier function in injured intestinal mucosa. Physiol Rev. (2007) 87:545–64. 10.1152/physrev.00012.2006 17429041

[66] Patel A Mamtani M Dibley MJ Badhoniya N Kulkarni H Therapeutic value of zinc supplementation in acute and persistent diarrhea: a systemic review. PLoS ONE. (2010) 5:e10386 10.1371/journal.pone.0010386 20442848 PMC2860998

[67] Hu CH Xiao K Song J Luan ZS Effects of zinc oxide supported on zeolite on growth performance, intestinal microflora and permeability, and cytokines expression of weaned pigs. Anim Feed Sci Technol. (2013) 181:65–71. 10.1016/j.anifeedsci.2013.02.003

[68] Li K Xiao Y Chen J He X Yang H. Microbial composition in different gut locations of weaning piglets receiving antibiotics. Asian Australas J Anim Sci. (2017) 30:78–84. 10.5713/ajas.16.0285 27383806 PMC5205595

[69] Hyun Y Ellis M Riskowski G Johnson RW. Growth performance of pigs subjected to multiple concurrent environmental stressors. J Anim Sci. (1998) 76:721–7. 10.2527/1998.763721x 9535330

[70] Sanz Fernandez MV Pearce SC Gabler NK Patience JF Wilson ME Socha MT. Effects of supplemental zinc amino acid complex on gut integrity in heat-stressed growing pigs. Animal. (2014) 8:43–50. 10.1017/S1751731113001961 24229744

[71] Pluske JR Turpin DL Kim J-C. Gastrointestinal tract (gut) health in the young pig. Anim Nutr. (2018) 4:187–96. 10.1016/j.aninu.2017.12.004 30140758 PMC6104527

[72] Balachandar J Nyachoti CM Husbandry practices and gut health outcomes in weaned piglets: a review. Anim Nutr. (2017) 3:205–11. 10.1016/j.aninu.2017.06.002 29767154 PMC5941228

[73] Furugouri K Kawabata A. Iron absorption in nursing piglets. J Anim Sci. (1975) 41:1348–54. 10.2527/jas1975.4151348x 1194120

[74] Lönnderdal B Development of iron homeostasis in infants and young children. Am J Clin Nutr. (2017) 106:1575S−80S. 10.3945/ajcn.117.155820 29070561 PMC5701726

[75] Cooper C Nonnecke EB Lönnderdal B Murray J The lactoferrin receptor may mediate the reduction of eosinophils in the duodenum of pigs consuming milk containing recombinant human lactoferrin. Biometals. (2014) 5:1031–38. 10.1007/s10534-014-9778-8 25085595

[76] Liao Y Lopez V Shafizadeh TB Halsted CH Lönnerdal B. Cloning of pig homologue of the human lactoferrin receptor: expression and localization during intestinal maturation in piglets. Comp Biochem Physiol A Mol Integr Physiol. (2007) 148:584–90. 10.1016/j.cbpa.2007.08.001 17766154 PMC2265088

[77] Rincker MJ Hill GM Link JE Rowntree J. Effects of dietary iron on supplementation on growth performance, hematological status, and whole-body mineral concentrations of nursery pigs. J Anim Sci. (2004) 82:3189–97. 10.2527/2004.82113189x 15542465

[78] Hansen SL Trakooljul N Liu HC Moeser AJ Spears JW Iron transporters are differentially regulated by dietary iron, and modification are associated with changes in manganese metabolism in young pigs. J Nutr. (2009) 139:1474–9. 10.3945/jn.109.105866 19535423

[79] Blachier F Vaugelde P Robert V Kibangou B Canonne-Hergaux F Delpal S. Comparative capacities of the pig colon and duodenum for luminal iron absorption. Can J Physiol Pharmacol. (2007) 85:185–92. 10.1139/Y07-007 17487259

[80] Dostal A Lacroix C Pham VT Zimmermann MB Del'homme C Bernalier-Donadille A Iron supplementation promotes gut microbiota metabolic activity but not colitis markers in human gut microbiota-associated rats. Br J Nutr. (2014) 111:2135–45. 10.1017/S000711451400021X 24555487

[81] Li Y Hansen SL Borst LB Spears JW Moeser AJ. Dietary iron deficiency and oversupplementation increase intestinal permeability, ion transport, and inflammation in pigs. J Nutr. (2016) 146:1499–505. 10.3945/jn.116.231621 27358414 PMC4958291

[82] Lönnderdal B Excess iron intake as a facto in growth, infections, and development of infants and young children. Am J Clin Nutr. (2017) 106:1681S–987S. 10.3945/ajcn.117.156042 29070544 PMC5701711

[83] Garcia-Aranda J Wapnir R Lifshitz F. *In vivo* intestinal absorption of manganese in the rat. J Nutr. (1983) 113:2601–7. 10.1093/jn/113.12.2601 6655516

[84] Keen C Lönnerdal B Hurley LS The role of zinc, manganese, and copper in rumen metabolism and immune function: a review article. In: Freiden E editor. Biochemistry of the Essential Ultratrace Elements. New York, NY: Plenum (1984). pp. 89–132.

[85] Li S Luo X Liu B Crenshaw TD Kuang X Shao G. Use of chemical characteristics to predict the relative bioavailability of supplemental organic manganese sources for broilers. J Anim Sci. (2004) 82:2352–63. 10.2527/2004.8282352x 15318735

[86] Creech BL Spears JW Flowers WL Hill GM Lloyd KE Armstrong TA. Effect of dietary trace mineral concentration and source (inorganic vs. chelated) on performance, mineral status, and fecal mineral excretion in pigs from weaning through finishing. J Anim Sci. (2004) 82:240–7. 10.2527/2004.8272140x 15309962

[87] Sawyer JT Tittor AW Apple JK Morgan JB Maxwell CV Rakes LK. Effects of supplemental manganese on performance of growing-finishing pigs and pork quality during retail display. J Anim Sci. (2007) 85:1046–53. 10.2527/jas.2006-262 17178808

